# suPAR to Risk-Stratify Patients With Malaria

**DOI:** 10.3389/fimmu.2022.931321

**Published:** 2022-06-10

**Authors:** Veselina Stefanova, Valerie M. Crowley, Andrea M. Weckman, Kevin C. Kain

**Affiliations:** ^1^ Department of Laboratory Medicine and Pathobiology, University of Toronto, Toronto, ON, Canada; ^2^ Sandra A. Rotman (SAR) Laboratories, Sandra Rotman Centre for Global Health, University Health Network-Toronto General Hospital, Toronto, ON, Canada; ^3^ Department of Experimental Therapeutics, University Health Network-Toronto General Hospital, Toronto, ON, Canada; ^4^ Faculty of Medicine, University of Toronto, Toronto, ON, Canada; ^5^ Tropical Disease Unit, Division of Infectious Diseases, Department of Medicine, University of Toronto, Toronto, ON, Canada

**Keywords:** soluble urokinase-type plasminogen activator receptor (suPAR), prognostic marker, triage, risk stratification, malaria

## Abstract

Severe malaria (SM) is a leading cause of global morbidity and mortality, particularly in children in sub-Saharan Africa. However, existing malaria diagnostic tests do not reliably identify children at risk of severe and fatal outcomes. Dysregulated host immune and endothelial activation contributes to the pathogenesis of SM. Current research suggests that measuring markers of these pathways at presentation may have clinical utility as prognostic indicators of disease progression and risk of death. In this review, we focus on the available evidence implicating soluble urokinase-type plasminogen activator receptor (suPAR) as a novel and early predictor of severe and fatal malaria and discuss its potential utility for malaria triage and management.

## 1 Introduction

Malaria remains a primary cause of childhood illness and death in sub-Saharan Africa (1). Malaria rapid diagnostic tests (RDTs) are overwhelmingly the diagnostic tool used in malaria-endemic countries to confirm suspected cases. Although RDTs are less sensitive than microscopy or nucleic acid amplification techniques, they are fast, inexpensive, and a low-expertise method to quickly diagnose *Plasmodium falciparum* and *Plasmodium vivax* infections. Regardless of the method used to detect malaria parasites, no current diagnostic test can reliably predict which children will progress to severe or fatal disease. Dysregulated host immune and endothelial activation contributes to the pathogenesis of severe malaria (SM) (2). Host-based prognostic biomarkers, especially if incorporated into RDTs, have the potential to improve risk stratification and outcome of children with malaria (2–6).

Soluble urokinase-type plasminogen activator receptor (suPAR) is an indicator of immune and endothelial activation and plays an important role in processes including cell migration, adhesion, and chemotaxis. Elevated suPAR levels have been linked to poor prognosis in various infections including HIV-1, tuberculosis, and sepsis. Emerging evidence also suggests that increased circulating levels of suPAR are associated with disease severity and mortality in children with malaria. Here, we report an overview of urokinase/urokinase-type plasminogen activator receptor (uPA/uPAR) biology, review what is known about suPAR in pediatric malaria, and discuss the ability of suPAR to predict disease outcome and its potential as a druggable target. We summarize putative mechanisms through which suPAR may contribute to the pathogenesis of malaria. Lastly, we outline the need for research investigating the causal role of suPAR in malaria and the evaluation of a currently available suPAR point-of-care test as a potential triage tool for severe malaria in prospective trials.

## 2 Structure and Function of suPAR

**Figure 1:**
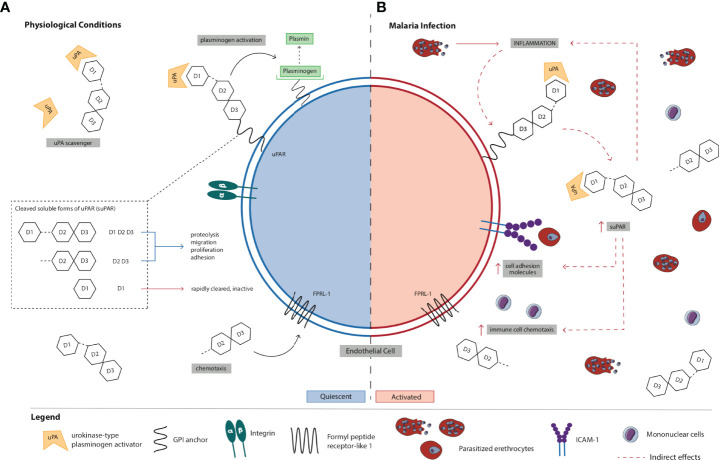
Schematic representation of the **(A)** normal physiological role of suPAR in inflammation and immune activation vs. **(B)** proposed mechanism of action of suPAR in the pathogenesis of severe malaria. **(A)** uPAR is a three-domain (D1, D2, and D3) GPI-anchored protein expressed on the cell surface of immune, endothelial, epithelial, and smooth muscle cells. Full-length suPAR is involved in a variety of cellular processes including proteolysis, migration, proliferation, and adhesion through its interactions with ECM proteins such as integrins and vitronectins as well as other receptors. Also, suPAR is a regulator of the plasminogen activation system since it can scavenge and bind the serine protease uPA in the ECM thereby competitively inhibiting uPAR. Cleaved suPAR interacts with FPRL-1 to induce chemotaxis of immune cells (e.g., monocytes, neutrophils). suPAR D1 is rapidly cleared from circulation and is biologically inactive. suPAR is released into circulation during infection, and therefore, the circulating concentrations of suPAR reflect the extent of immune activation and inflammation in an individual. Under normal physiological conditions, low levels of circulating suPAR are detected in the healthy population. **(B)** During *P. falciparum* infection, PEs bind to and sequester in microvascular endothelial cells. Recognition and binding of parasite products (e.g., PfGPI) to Toll-like receptors on monocytes and ECs activates them to produce and secrete pro-inflammatory cytokines (e.g., TNF-α) and chemokines, and there is upregulation of the expression of cell-adhesion molecules on endothelial cells (e.g., ICAM-1) to which PEs bind. Increased production of pro-inflammatory cytokines/chemokines and immune activation stimulate uPAR-expressing cells (e.g., monocytes) to produce and secrete suPAR. Initially, increased local levels of suPAR in children with malaria may promote protective innate immune responses in the host defense by promoting the recruitment of immune cells (e.g., neutrophils and monocytes) to acute sites of infection *via* its chemotactic effector functions. Through its interactions with other ECM proteins and receptors (e.g., integrins), suPAR can also trigger downstream signaling resulting in the upregulation and expression of adhesion molecules (e.g., ICAM-1). In the absence of early treatment, parasite numbers continue to increase and PEs further accumulate and sequester in microvascular ECs due to the upregulated expression of adhesion molecules. As a result, the production and secretion of pro-inflammatory cytokines/chemokines are exacerbated, ultimately resulting in a systemic increase in inflammation and immune activation. Elevated circulating suPAR may amplify the release of cytokines/chemokines and the recruitment of immune cells, which contributes to sustained systemic inflammation and immune activation. Excessive inflammation in malaria could also trigger biological processes leading to marked increases in circulating suPAR. Although further studies are needed to elucidate its role, suPAR may contribute to enhanced chemokine and cytokine secretion that culminates in endothelial dysfunction, multiorgan failure, and death in children with malaria. High circulating concentrations of suPAR in children with malaria may therefore reflect excessive activation of immune cells, adhesion of PEs at sites of inflammation, disturbances in hemostasis, or a combination of these. Collectively, suPAR may be involved in a positive feedback loop that results in high levels of local and systemic immune activation and inflammation through its own pro-inflammatory properties and by activating and recruiting other chemokines and cytokines *via* chemotaxis. Also, the pathobiology of severe falciparum malaria is associated with upregulation of coagulation pathways. Elevated suPAR levels may indirectly exert pro-coagulant effects by enhanced binding to uPA and competitive inhibition of membrane-bound uPAR thereby indirectly inhibiting uPA-dependent fibrinolysis or by stimulating activation of coagulation mechanisms *via* intrinsic and extrinsic pathways. Elevated levels of suPAR in children with malaria may reflect the degree to which some or all of these processes occur. EC, endothelial cell; ECM, extracellular matrix; *P. falciparum*, *Plasmodium falciparum*; PfGPI, GPI, *Plasmodium falciparum* glycosylphosphatidylinositol; GPI, glycosylphosphatidylinositol; ICAM-1, intercellular adhesion molecule-1; PE, parasitized erythrocyte; SM, severe malaria; suPAR, soluble urokinase-type plasminogen activator receptor; uPA, urokinase or urokinase-type plasminogen activator; uPAR, urokinase-type plasminogen activator receptor.

uPAR is a glycosylphosphatydilinositol (GPI)-anchored protein that contains three homologous domains (D1, D2, and D3) and is expressed on the surface of various cell types including immune, endothelial, epithelial, and smooth muscle cells (7, 8). suPAR is the soluble form of the membrane-bound receptor uPAR that is released into circulation (9) (**Figure 1**). Three soluble forms of uPAR are produced by cleavage at the GPI anchor and/or linker region connecting D1 and D2: suPAR D1–D3 (full-length suPAR), suPAR D2D3 (cleaved suPAR), and suPAR D1. Full-length suPAR is involved in various cellular processes including proteolysis, migration, adhesion, and proliferation since it can interact with other extracellular matrix (ECM) proteins and receptors (e.g., integrins and vitronectins) (10, 11). Also, this soluble form of uPAR is a scavenger of urokinase-type plasminogen activator (uPA; also known as urokinase), the ligand for membrane-associated uPAR, in the ECM; thus, full-length suPAR can competitively inhibit uPAR (12). In contrast, cleaved suPAR is primarily involved in the chemotaxis of immune cells (e.g., monocytes and neutrophils) (13). Unlike these active forms of suPAR, suPAR D1 has no known biological functions and is rapidly cleared from circulation (14).

### 2.1 The uPA/uPAR System at the Intersection of Inflammation, Fibrinolysis, and Coagulation

uPA, a serine protease, and its cellular receptor uPAR exhibit pleiotropic functions during both physiological and pathological processes. The uPA/uPAR system mediates numerous critical cellular functions including extracellular proteolysis, chemotaxis, cell adhesion, vascular homeostasis, and tissue remodeling and repair (10, 15–17).

The uPA/uPAR system has a central role in fibrinolysis and the modulation of host inflammatory and immune responses (18, 19). Binding of uPA to membrane-bound uPAR catalyzes the activation of plasminogen to plasmin, an enzyme critical for the degradation of fibrin (20). Notably, uPA-mediated plasminogen activation is implicated in various processes requiring cell migration, which is an essential event in both physiological and pathological processes (e.g., cell recruitment, wound healing, and angiogenesis) (21–24).

The uPA molecule is directly involved in mechanisms of migration, adhesion, and chemotaxis (16). Importantly, uPA can regulate many of these processes independent of uPAR binding. Unlike uPA, which can directly mediate fibrinolysis by activating plasminogen, uPAR is thought to indirectly participate in local fibrinolysis through its chemotactic activity (25). Full-length suPAR, which contains the D1 uPA-binding domain, can bind many of the same ligands (e.g., uPA, integrins, and vitronectin) as membrane-bound uPAR (12, 26). Therefore, suPAR–integrin interactions may trigger downstream signaling events. In addition, increased production of suPAR may disrupt uPA/uPAR-dependent proteolysis and/or alter cell signaling pathways since suPAR competes with membrane-bound uPAR for uPA in the ECM (27). In effect, suPAR may be a negative regulator of uPA/uPAR-dependent plasminogen activation and contribute to inhibited fibrinolysis, i.e., suPAR may indirectly exert pro-coagulant functions. Thus, suPAR may induce and modulate uPAR-dependent cell signaling responses and the processes catalyzed by these molecules and their interaction with membrane-associated uPAR (16). In contrast, cleaved suPAR (suPAR D2D3), which lacks the D1 binding domain, is mainly involved in chemotaxis since it can activate formyl peptide receptor-like 1 (FPRL-1) thereby promoting the immune response. The production of this form of suPAR by activated neutrophils in sites of acute inflammation may contribute to the recruitment of monocytes to these sites during infection (28).

#### 2.1.1 The uPA/uPAR System Promotes Inflammation and Immune Activation

In addition to its role in fibrinolysis, the uPA/uPAR system participates in inflammatory and immune activation pathways (**Figure 1A**). uPAR anchors uPA at the cell surface which favors ECM degradation and regulates cell migration, adhesion, and proliferation, thereby influencing the development of inflammatory and immune responses (15, 21). Since membrane-tethered uPAR lacks transmembrane and intracellular domains, it requires interactions with other coreceptors and proteins to induce cell signaling and mediate cytoskeletal reorganization (10, 29). uPAR on the cell surface interacts with integrin β1, β2, and β3 family members and G-protein-coupled receptors (GPRs) including FPRL-1 to modulate cell adhesion and migration (10, 16, 30). Membrane-bound uPAR is involved in the recruitment of leukocytes to acute sites of inflammation *via* integrated mechanisms including proteolysis, adhesion, migration, and mitogenesis (21, 22). The functional interaction between uPAR and integrins is believed to be critical for leukocyte recruitment and activation (21). In addition, membrane-bound uPAR interacts with FPRL-1 to exert chemotactic effector functions, which promotes the recruitment and activation of immune cells (e.g., monocytes, neutrophils, macrophages) during immune and inflammatory responses (16). Vitronectin, an extracellular protein, is another important uPAR ligand that promotes cell adhesion and migration (8). Vitronectin is at the intersection of the uPA/uPAR and integrin systems since it can bind both uPAR and integrins, and integrin-bound vitronectin is required for uPAR (31, 32). In addition, uPAR regulates integrin activity and uPAR-bound uPA enhances uPAR binding to vitronectin, which highlights the importance of the uPA/uPAR system in coordinating cell–cell and cell–ECM interactions (21, 29). uPAR and its partners cross-regulate each other and there is considerable cross-talk between these pathways (32).

### 2.2 uPAR/suPAR and Endothelial Dysfunction

Dysregulated host endothelial activation is a key pathophysiological feature in *P. falciparum* infection (2). uPAR and suPAR have previously been linked with endothelial dysfunction (33–35). uPAR is expressed on endothelial cells and upregulated during endothelial activation (36, 37). Post-mortem tissues of patients with cerebral malaria (CM) showed upregulated expression of uPAR on endothelial cells limited to CM-associated lesions, implicating uPAR in blood–brain barrier disruption and immunologic injury during CM (38). The authors also suggested that uPAR may be an additional adhesion molecule for parasitized erythrocytes (PEs) on activated endothelial cells since uPAR reactivity exclusively co-localized to lesions with PEs sequestered in cerebral microvasculature. suPAR levels also positively correlate with circulating markers of endothelial dysfunction (39, 40). The uPA/uPAR system has been suggested to contribute to the pathogenesis of SM by influencing platelet sequestration and/or activating the endothelium (37, 38, 41, 42). Upregulated expression of cellular adhesion molecules (e.g., ICAM-1) on endothelial cells promotes sequestration of PEs to the vascular endothelium *in vivo* (43, 44). Interactions between endothelial cells and immune cells stimulate the release of suPAR, suggesting a potential link between elevated levels of suPAR and inflammatory processes in the microvascular endothelium (45). Given the role of suPAR in modulating immune responses and cell adhesion, it is possible that suPAR may contribute to endothelial dysfunction in SM by inducing intracellular signaling cascades that lead to upregulated expression of cellular adhesion molecules on the surface of microvascular endothelial cells and/or promoting pro-inflammatory cytokine production, which may also contribute to the upregulation of host adhesion receptors.

### 2.3 uPA, uPAR, and suPAR Are Upregulated During Infection

The importance of the uPA/uPAR system in modulating immune and inflammatory responses during infection has been well documented. uPAR expression is upregulated on endothelial and hematopoietic cells during bacterial infection and in response to pro-inflammatory cytokines [e.g., tumor necrosis factor alpha (TNF-α), interferon gamma (IFN-γ), and interleukin 2 (IL-2)] (25). For example, patients with melioidosis have upregulated uPAR expression, and in the experimental model of *Burkholderia pseudomallei* melioidosis, uPAR knockout mice had reduced neutrophil migration to the primary site of infection and increased bacterial growth and organ inflammation (46). These findings indicate that uPAR deficiency may result in impaired host innate immune responses to infection. *In vitro*, uPAR-deficient macrophages and granulocytes had impaired phagocytosis of *B. pseudomallei*. Similar findings from other animal models show that uPAR deficiency in mice impairs the recruitment of neutrophils and leukocytes (22, 47). *In vitro*, antisense blockade of uPAR leads to impaired leukocyte migration (48). uPA-knockout mice have impaired recruitment of mononuclear cells during infection *in vivo* (49). Furthermore, the uPA/uPAR system is involved in the activation and differentiation of T cells; uPA and uPAR are upregulated during T-cell activation (19). In response to *Pseudomonas aeruginosa* infection, uPA- and uPAR-knockout mice have impaired lymphocyte recruitment to the lung, which suggests that uPA/uPAR may promote T-cell effector functions at the sites of infection (22). Collectively, these findings show that uPA and uPAR modulate host defense in response to infection and are important for protective immunity. The role of suPAR in innate immune responses *in vivo* is relatively unexplored. Evidence suggests that suPAR may elicit the recruitment and activation of pro-inflammatory cells (i.e., monocytes and neutrophils) (28). However, as with other innate immune responses, dysregulated suPAR production during infection has the potential to mediate disease pathobiology.

#### 2.3.1 Dysregulation of the uPA/uPAR System Is Linked to Pathology

Upregulated uPA, uPAR, and suPAR are linked with various pathological conditions (50–55). In patients with systemic inflammation and cirrhosis, circulating suPAR levels are related to immune activation and function as a marker of poor clinical outcomes (56, 57). *In vivo*, suPAR may stimulate the recruitment and activation of leukocytes, which promotes inflammation and immune activation (28). Excessive inflammation is an established mechanism contributing to tissue damage and organ dysfunction in multiple infections (58–60). Thus, it is not unexpected that dysregulation of the uPA/uPAR system and suPAR, which promotes inflammation and immune activation, may mediate host pathobiology. Evidence from a preclinical study of HIV-1 supports this notion. In cell culture, suPAR expression and release was upregulated in lymphoid organs during HIV-1 infection (53). Cleaved suPAR in HIV-infected cells inhibited chemotaxis and induced virus expression, suggesting that suPAR contributes to dysregulated immune activation and pathogenesis in HIV infection. Considerable evidence from clinical studies also supports a link between suPAR and the pathophysiology and clinical outcomes of various life-threatening infections, including malaria (40, 61, 62).

## 3 suPAR Is a Marker of Disease Progression and Poor Prognosis in Infectious Diseases

The prognostic value of suPAR has previously been documented in infectious diseases of variable origin (bacterial, viral, and parasitic) including tuberculosis, bacteremia and/or sepsis, meningitis, hepatitis B and C, HIV-1, hemorrhagic fever, hantavirus, malaria, and most recently coronavirus disease 2019 (COVID-19) (63–72). Across multiple infectious diseases, suPAR levels are elevated and predict the severity of illness and poor clinical outcomes.

In patients with HIV-1 infection, suPAR is associated with disease severity, mortality risk, and ineffective immune recovery (54, 68). Elevated plasma suPAR levels (both intact and cleaved suPAR) have been reported to reflect immune activation and independently predict mortality outcome in HIV-1-infected patients (68). In a study of critically ill children with sepsis, circulating suPAR levels were significantly higher in non-survivors compared with survivors, and suPAR was a reliable indicator of ICU mortality (73). Consistent with these findings, suPAR was an excellent predictor of mortality outcome in patients with hemorrhagic fever (69). Similarly, patients with meningitis have elevated cerebrospinal fluid suPAR levels, and increased suPAR is a strong predictor of a fatal outcome (61). suPAR is also associated with clinical severity and case fatality in COVID-19 (40, 72).

In a study of patients with symptoms of COVID-19, suPAR levels were significantly higher in patients who developed severe and critical illness compared with those who were moderately ill, and it was shown that suPAR cutoffs could be used to risk-stratify patients (74, 75). In hospitalized COVID-19 patients, suPAR was predictive of in-hospital acute kidney injury (AKI) and the need for dialysis (76). At least in COVID-19, suPAR has been shown to be an early indicator of endogenous alarmins such as IL-1a, and its levels increase earlier than other biomarkers of disease progression, such as CRP, IL-6, and D-dimers (76, 77). It is this early warning, sentinel-like property of suPAR that positions it as a useful tool for patient triage and has been applied as such in at least three studies of COVID-19. suPAR point-of-care (POC) testing was used to triage and discharge patients with COVID-19 symptoms early from the emergency department (78).

Another set of studies used suPAR POC testing to quantify suPAR levels, risk-stratify patients to identify those at higher risk of respiratory failure or death, and provide early targeted treatment for these individuals with a drug intervention (anakinra) (79, 80).

## 4 The Prognostic Role of suPAR in Malaria

### 4.1 suPAR in Children With Malaria

Only a few studies to date have examined the prognostic role of suPAR in malaria (**Supplementary Table 1**). Limited data have linked elevated suPAR levels with disease severity and outcome in pediatric malaria. In a study of African children with acute *P. falciparum* malaria, Perch et al. found that serum suPAR levels at study inclusion were associated with parasite density and children with the highest parasitemia had significantly higher suPAR levels than children who had lower parasitemia or had a negative blood film (81). Of note, suPAR levels were significantly reduced in all malaria-positive children 7 days following initiation of antimalarial treatment. The most marked reduction in suPAR levels following treatment was in the group with the highest parasitemia where suPAR decreased to almost half its level at inclusion; however, this level was still significantly higher than the suPAR levels reported in all other groups on day 7. The authors suggest that elevated suPAR levels in children with high parasitemia may normalize over a longer treatment period. Similarly, Ostrowski et al. reported significantly higher plasma suPAR concentrations in malaria-positive children compared with those who were malaria-negative and healthy controls (82). Among the malaria-positive children, the highest suPAR levels were in children who had complicated diseases and in those who died. suPAR positively correlated with parasitemia and increased plasma suPAR concentration at admission was associated with a fatal outcome.

Contrary to the findings of Ostrowski et al., elevated suPAR levels were not associated with poor outcome in Cameroonian children with falciparum malaria (83). Instead, a gradual trend in increasing plasma suPAR levels was observed between children with asymptomatic malaria (AM) and those with uncomplicated malaria (UM) as well as between children with SM and those with CM. suPAR was excellent at discriminating between children with AM and those with UM with an area under the receiver operating characteristic curve (AUROC) of 0.958. However, a strong association with SM was not observed. suPAR levels were not informative in differentiating between severe (non-cerebral) and cerebral malaria. The lack of relationship between suPAR and SM reported in this study is unknown but may reflect, at least in part, the absence of fatal cases in the study population. In a study of Beninese children with *P. falciparum* infection, plasma suPAR levels were associated with malaria severity, coma, and mortality (84). suPAR was significantly higher in children with SM [CM/severe non-cerebral malaria (SNCM)] than in children with UM. Elevated suPAR levels were also associated with the presence of coma. suPAR was significantly higher in children with SM (CM/SNCM) who died compared with those who survived. However, suPAR was not a strong discriminator between SM subtypes (CM vs. SNCM). When compared with other inflammatory, angiogenic, and vascular markers, suPAR had relatively low prognostic accuracy in discriminating between UM vs. CM/SNCM, coma vs. no coma, and fatal vs. non-fatal cases with AUROC <0.70 for each analysis.

In summary, the available studies were largely underpowered to assess mortality outcomes. Therefore, the current evidence base is inadequate to determine whether suPAR levels are clinically useful as prognostic predictors in pediatric malaria. Additional larger prospective studies that enroll consecutive children with a broad disease severity spectrum are required to determine the utility of suPAR to risk-stratify children with malaria.

### 4.2 suPAR in Adults With Severe Malaria

Evidence for the prognostic role of suPAR in adults with malaria infection is limited. In a study of Bangladeshi adults with *P. falciparum* infection and AKI, increased suPAR levels were associated with increasing AKI severity, and levels at admission predicted a later requirement for renal replacement therapy (62). However, suPAR concentrations were not significantly different between survivors and non-survivors with AKI in SM. The authors posited that the lack of association between suPAR levels and mortality in this study may reflect the limited spectrum of malaria severity in their cohort (i.e., all patients had severe illness) and that including patients with UM might confirm the potential link between suPAR levels and mortality outcome in adults with SM. Alternatively, it is possible that elevated suPAR levels in adults with SM may not predict an increased risk of death or do so, albeit, to a lesser extent than in children. This could be expected since age is a known risk factor for fatal outcome in patients with SM and the risk of mortality is disproportionately higher in children under 5 years old (85).

### 4.3 suPAR in Malaria in Pregnancy

To date, there is only one study that has evaluated the prognostic role of suPAR in malaria in pregnancy (71). In their study, Ostrowski et al. investigated the relationship between suPAR levels and fetal outcome in pregnant African women. Plasma suPAR concentrations were measured in maternal (at enrolment) and cord (at delivery) samples in women with histology-confirmed placental malaria (active or past infection) and women with no history of malaria infection. They found that maternal suPAR levels were significantly higher in women with active malaria infection compared with women with past infection or non-infected. Cord suPAR levels did not differ across placental histology groups. Maternal suPAR was also positively correlated with maternal peripheral parasitemia. Importantly, in women with active infection, elevated maternal suPAR independently predicted low birth weight. Both maternal and cord suPAR levels were not associated with stillbirth in any of the histology groups. These findings suggest that maternal suPAR levels are potentially associated with adverse birth outcomes in women with malaria. However, cord suPAR levels were not associated with birth weight after adjusting for gestational age, suggesting that cord suPAR is less influenced by maternal malaria infection. The role of suPAR in malaria in pregnancy needs to be further evaluated in prospective studies with longitudinal sampling across pregnancy and assessment of additional adverse birth outcomes (e.g., preterm birth).

## 5 A Mechanistic Role for suPAR in Malaria Pathology?

Given that dysregulated endothelial and immune activation contributes to the pathogenesis of severe falciparum malaria, we propose a model of how suPAR may be a mediator of these pathways (**Figure 1B**). The limited preclinical data that exist suggest a role of the uPA/uPAR pathway in the pathogenesis of malaria. uPAR-deficient mice infected with *Plasmodium berghei* have reduced mortality, attenuated thrombocytopenia, absent platelet trapping, enhanced leukocytosis, and reduced apoptosis compared with wild-type mice (41). Evidence that uPA/uPAR deficiency in *P. berghei*-infected mice attenuates disease severity and delays mortality *in vivo* suggests that attenuation of this pathway (including suPAR) might be beneficial. In cell culture experiments, uPA binds to human erythrocytes infected with mature forms of *P. falciparum* and is required for the rupture of erythrocytes and the release of merozoites, which suggests that uPA may be an additional adhesion molecule for PEs (86). The only evidence to date that implicates the uPA/uPAR pathway to human malaria pathogenesis comes from a post-mortem study of patients with CM that reported increased expression of uPAR on macrophages, microglial cells, astrocytes, and endothelial cells in CM-associated lesions, suggesting that uPAR may contribute to blood–brain barrier disruptions and immunopathology in CM (38).

Additional studies are required to determine whether the uPA/uPAR pathway plays a mechanistic role in the pathogenesis of malaria. Antibodies or small molecular inhibitors that prevent uPAR cleavage or that specifically bind and sequester suPAR from circulation could provide further insights (55, 87). Experimental evidence from a murine model of focal segmental glomerulosclerosis (FSG) indicates that circulating levels of suPAR can contribute to the pathogenesis of FSG and that administration of blocking uPAR antibodies improves suPAR-induced kidney damage (55). If suPAR directly contributes to the pathogenesis of SM, this pathway could represent a potential therapeutic target.

## 6 Conclusions

Prognostic/severity markers could facilitate the early recognition and treatment of children with impending severe malaria. Integrating these markers into POC RDT platforms, or added to current malaria RDTs, could transform community-based triage of pediatric malaria cases, especially in low-resource settings (3). This could inform individualized management and potentially reduce malaria deaths. The few available studies investigating the prognostic role of suPAR in children with malaria have been underpowered to robustly assess clinical outcomes (most have few or no fatal cases) and/or use limited measures of disease severity. Discrepancies in suPAR levels reported across studies in patients with malaria may reflect differences in the assay used to quantify suPAR (**Supplementary Table 1**) and may limit the comparability of suPAR findings. Alternatively, they could be explained by varying baseline circulating suPAR concentrations in different patient populations; differences in age, malaria exposure, and immunity status; other coinfections or different etiologies inducing changes in suPAR; and/or underlying nutritional deficiencies which may affect the levels of inflammation and immune activation and, thus, circulating suPAR concentrations (63, 68, 88–91). Future studies controlling for these factors and using standardized commercial kits to quantify suPAR would help to elucidate the potential relationship between suPAR levels and clinical outcomes (e.g., mortality) in patients with malaria. In addition, large prospective studies including children across a broad disease severity spectrum with adequate power to assess mortality outcomes are required to confirm the potential value of suPAR to risk-stratify children with malaria. These studies would need to determine and validate quantitative cutoffs that can accurately identify patients needing urgent care and parenteral artesunate treatment. Several testing platforms are available to quantify suPAR concentrations including turbidimetric immunoassays, sandwich ELISAs, and magnetic bead-based multiplex assays. Given that a POC test for suPAR already exists, prospective trials can be designed to assess its clinical utility and establish cutoffs to guide triage and management decisions (74). The validation of a POC test based on suPAR levels that accurately risk-stratifies children with malaria could have a direct impact for enhanced triage and management and improved health outcomes for children in resource-limited settings.

## Author Contributions

VS and VC conceived the ideas for preparing this review. AW created the figure and VS created the table. VS wrote the first draft of the manuscript. VC, AW, and KK wrote sections of the manuscript. All authors contributed to manuscript revision and read and approved the submitted version.

## Funding

This work was supported by the Canadian Institutes of Research (CIHR) Foundation grant (FDN-148439 to KK) and the Canada Research Chair Program (KK). This work was also supported by a Collaborative Research Agreement Grant from Intellectual Ventures/Global Good (KK), the Bill and Melinda Gates Foundation Trust through Intellectual Ventures/Global Good, and donations from Kim Kertland and the Tesari Foundation.

## Conflict of Interest

The authors declare that the research was conducted in the absence of any commercial or financial relationships that could be construed as a potential conflict of interest.

## Publisher’s Note

All claims expressed in this article are solely those of the authors and do not necessarily represent those of their affiliated organizations, or those of the publisher, the editors and the reviewers. Any product that may be evaluated in this article, or claim that may be made by its manufacturer, is not guaranteed or endorsed by the publisher.

## Abbreviations

AKI, acute kidney injury; CI, confidence interval; CM, cerebral malaria; ECM, extracellular matrix; ELISA; enzyme-linked immunosorbent assay; FPRL-1, formyl peptide receptor-like 1; FSG, focal segmental glomerulosclerosis; GPI, glycosylphosphatidylinositol; ICAM-1, intercellular adhesion molecule 1; IFN-γ, interferon gamma; IL-2, interleukin 2; RDT, rapid diagnostic test; SM, severe malaria; suPAR, soluble urokinase-type plasminogen activator receptor; TLR, Toll-like receptor; TNF-α, tumor necrosis factor alpha; UM, uncomplicated malaria; uPA, urokinase; uPAR, urokinase-type plasminogen activator receptor.
