# Supplementary material for: suPAR to Risk-Stratify Patients With Malaria
Source: Front Immunol. 2022 Jun 10;13:931321. doi: 10.3389/fimmu.2022.931321 (PMC9226448; doi:10.3389/fimmu.2022.931321)
Supplement: Supplementary file 1 [file Table_1.pdf]

Table 1. **Studies evaluating the prognostic value of soluble urokinase-type plasminogen activator receptor in patients with malaria.**

First author, publication date (ref)	Study design	Malaria diagnostic method	Participants	Period	Outcome variables	suPAR quantification	suPAR levels (ng/mL)	Main suPAR findings
Perch, 2004 (81)	Prospective (1:1 case to control)	Microscopy confirmed *P. falciparum* infection	87 African children with clinical symptoms of malaria. Three clinical groups based on parasite density: group 1, symptomatic malaria (> 20 parasites/200 leukocytes); group 2 (1 to 19 parasites/200 leukocytes); group 3, negative blood film. Controls (group 4): randomly selected healthy* children from same area<br><br>Serum samples at inclusion and day 7<br><br>No fatal cases	September 2002 to March 2003	Disease severity<br><br>Response to 7-day treatment	Customized double-sandwich ELISA	Median suPAR levels at inclusion: group 1: 6.51 ng/mL; group 2, 3.04 ng/mL; group 3: 3.26 ng/mL<br><br>Median suPAR levels on day 7: Group 1: 3.48 ng/mL; Group 2: 2.50 ng/mL; Group 3: 2.47 ng/mL	suPAR was associated with parasitemia was significantly higher in children with high parasitemia (group 1) compared to children with low parasitemia (group 2) or who had a negative blood film. suPAR levels in groups 2 to 4 were not significantly different from each other. suPAR levels decreased from inclusion to day 7 in all four groups, with the greatest reduction occurring in group 1.
Ostrowski, 2005 (82)	Prospective	Microscopy-confirmed infection with *Plasmodium* parasites	645 African children with clinical symptoms of malaria (478 malaria-positive, 167 malaria-negative); 14 healthy sex- and age-matched controls from same geographic area<br><br>Plasma samples at hospital admission<br><br>Malaria deaths: 11/478 (2.3%)	June to August of 2000 and 2001	Disease severity<br><br>Mortality	Customized double-sandwich ELISA	Median suPAR concentrations: Malaria-positive, 7.9; malaria-negative, 5.59; healthy controls, 3.94	Higher suPAR concentration in children with malaria compared to malaria-negative children. Highest suPAR concentrations in children who died or had complicated malaria. suPAR was a univariate predictor of increased mortality risk and disease severity.
Tahar, 2016 (83)	Prospective	Microscopy-confirmed *P. falciparum* infection	215 African children with clinical symptoms of malaria (n=135) and asymptomatic carriers (n=80). Four clinical groups: asymptomatic (AM), uncomplicated malaria (UM), severe malaria (SM), cerebral malaria (CM). 28 healthy French Caucasian controls.<br><br>Plasma samples at admission<br><br>No fatal cases	Not reported	Disease severity	Commercial double-sandwich ELISA kit (DuoSet® ELISA Development System, R&D Systems, Minneapolis, MN)	Median suPAR concentrations: controls, 3.95 ng/mL; AM, 3.72 ng/mL; UM, 6.56 ng/mL; SM, 7.85 ng/mL; CM, 7.98 ng/mL	Gradual increase in suPAR levels between children with AM to UM and SM to CM. suPAR was excellent at discriminating between patients with UM and AM (AUROC, 0.958); however, suPAR levels could not distinguish between children with SM and CM. suPAR was strongly predictive of SM/CM compared to UM.

AKI, acute kidney injury; ELISA, enzyme-linked immunosorbent assay; *P. falciparum*, Plasmodium falciparum; SM, severe malaria; suPAR, soluble urokinase-type plasminogen activator receptor; UM, uncomplicated malaria

*More than half (67%) of control group had positive blood film and received treatment

Table 1. **Studies evaluating the prognostic value of soluble urokinase-type plasminogen activator receptor in patients with malaria (continued).**

**First author, publication date (ref)**	**Study design**	**Malaria diagnostic method**	**Participants**	**Period**	**Outcome variables**	**suPAR quantification**	**suPAR levels (ng/mL)**	**Main suPAR findings**
Tornyigah, 2022 (84)	Prospective (cross-sectional)	Rapid diagnostic test for *P. falciparum* (DiaQuick-Malaria-P. falciparum-Cassette, Dialab; Hondastrasse, Austria)	339 African children with malaria (234 with SM, 105 with UM) . Among children with SM, 101 had cerebral malaria (CM), 133 had severe non-cerebral malaria (SNCM).<br><br>Plasma samples at admission<br><br>Malaria deaths: 40/339 (11.8%)	December 2017 to July 2019	Severity<br><br>Coma<br><br>Mortality	Commercial double-sandwich ELISA kit (DuoSet® ELISA Development System, R&D Systems, Minneapolis, MN)	Not reported in text	suPAR was higher in children with SM (CM/SNCM) vs. UM and in children with coma vs. no coma. Children who died had higher suPAR levels than those who survived. All deaths occurred in children with SM. suPAR did not have strong predictive accuracy for severity, coma, or death compared to other markers measured.
Plewes, 2014 (62)	Retrospective	Microscopy-confirmed *P. falciparum* infection	106 Bangladesh adults with severe malaria complicated with AKI (32 mild, 42 moderate, 32 severe); 31 patients with falciparum malaria but no AKI.<br><br>Plasma samples at admission<br><br>Malaria deaths: 53/137 (39%).	2003 to 2005 and 2006 to 2010	AKI severity<br><br>Renal replacement therapy requirement<br><br>Mortality	Commercial double-sandwich ELISA kit (suPARnostic™, Virogates, Copenhagen, Denmark)	Mean suPAR concentrations: survivors, 29.03; non-survivors, 25.07	suPAR increased with AKI severity and was higher in patients who later required renal replacement therapy. suPAR levels were not significantly higher in patients who died compared to those who survived.
Ostrowski, 2007 (71)	Retrospective	Microscopy-confirmed placental malaria infection	253 pregnant African women (malaria-positive:<br>65 actively infected, 67 past-infected; 121 malaria-negative).<br><br>Maternal plasma at admission, cord plasma at delivery<br><br>Stillbirths in malaria-positive women: 12/132 (9.1%)	Women giving birth between January 1, 1996, and July 31, 1997	Fetal outcome	Customized double-sandwich ELISA[a]	Median maternal suPAR levels: actively infected, 3.93; past-infected, 2.67; non-infected, 2.78<br><br>Median cord suPAR levels: actively infected, 3.0; past-infected, 3.0; non-infected, 3.0	Maternal suPAR was higher in actively infected women compared to non-infected and past infected women. Cord suPAR was comparable across all groups. In actively infected women, maternal. suPAR was an independent predictor of low birth weight. Maternal and cord suPAR were not associated with stillbirth in any groups.

AKI, acute kidney injury; ELISA, enzyme-linked immunosorbent assay; *P. falciparum*, Plasmodium falciparum; SM, severe malaria; suPAR, soluble urokinase-type plasminogen activator receptor; UM, uncomplicated malaria
*More than half (67%) of control group had positive blood film and received treatment
